# Metabolic Profile and Mycoherbicidal Activity of Three *Alternaria alternata* Isolates for the Control of *Convolvulus arvensis*, *Sonchus oleraceus*, and *Xanthium strumarium*

**DOI:** 10.3390/pathogens10111448

**Published:** 2021-11-07

**Authors:** Nesma Abdessemed, Alessia Staropoli, Nadjia Zermane, Francesco Vinale

**Affiliations:** 1 Department of Botany, Ecole Nationale Supérieure d’Agronomie (ENSA, ex. INA), 16200 Algiers, Algeria; nesma.abdessemed@gmail.com; 2 Department of Agricultural Sciences, University of Naples Federico II, 80055 Portici, Italy; alessia.staropoli@unina.it; 3 Institute for Sustainable Plant Protection, National Research Council (CNR), Via Università, 80055 Portici, Italy; 4 Faculty of Sciences, University of Algiers, 16002 Algiers, Algeria; nadjiazermane@gmail.com; 5 Department of Veterinary Medicine and Animal Productions, University of Naples Federico II, 80137 Naples, Italy

**Keywords:** metabolomics, mass spectrometry, tenuazonicacid, SMTP-7

## Abstract

*Alternaria alternata* isolates C1, S1, and X3 were isolated respectively from the weeds *Convolvulus arvensis*, *Sonchus oleraceus*, and *Xanthium strumarium* in Algiers during 2016 and identified by morphological and molecular analyses. The aim of this investigation was to chemically characterize the exometabolome of these fungi and to evaluate the myco-herbicidal potential of their culture filtrates, crude extracts, or fractions towards target weeds. Results revealed a great heterogeneity in the biochemical profiles of the exometabolome with the remarkable presence of two compounds: tenuazonic acid (TeA) and triprenyl phenol-7 (SMTP-7). To the best of our knowledge, SMTP-7—found in all the isolates—as well as 12-methoxycitromycin detected in the culture filtrate of isolate C1, have never been reported to be produced by *A. alternata*. Some fractions of isolates C1 and S1 showed symptoms (necrosis and chlorosis) on the detached leaves of *C. arvensis* and *S. oleraceus*, respectively with up to 100% phytotoxic effect at low concentration. In conclusion, biochemical characterization revealed great difference of C1, S1, and X3 exometabolome that is likely to explain the difference in their phytotoxic activity. Some fractions (d_1_, e_1_, h_1_, i_1_, a_2_, and f_2_) of isolates C1 and S1 of *A. alternata* caused severe necrosis and chlorosis on the injured detached leaves of *C. arvensis* and *S. oleraceus,* respectively.

## 1. Introduction

*Alternaria* is a cosmopolitan fungal genus with 275 species [1], widely distributed in soil and organic matter. It includes saprophytic, endophytic, and pathogenic species [2,3]. *Alternaria* spp.can produce a variety of secondary metabolites [4]. In particular, at least 268 compounds have been reported in the past few decades that can be grouped into several categories, including nitrogen-containing compounds, steroids, terpenoids, pyranones (pyrones), quinones, and phenolics [2]. Many of these secondary metabolites are either mycotoxins or phytotoxins [5]. *Alternaria* spp. produces more than 70 mycotoxins [6], several species produce also various phytotoxins that are host-specific (HSTs) and non-host-specific (nHSTs) depending upon their physiological and morphological stages [6,7]. On the other hand, several studies demonstrated that metabolite profiling is a reliable tool to characterize and differentiate plant pathogenic fungi [8]. Microbial metabolites can be exploited as natural herbicides [9,10] and their use might be a promising alternative approach to designing bio formulates [11,12]. Their exploitation can also solve the problems of restrictions of living microorganisms such as: limitation of marketing, storage, difficulty of application, time of action, viability, influence of environmental conditions, etc. [13,14]. Several studies have been conducted to assess the herbicidal activity of fungal metabolites on weeds [15,16]. In this contest, some *A. alternata* compounds showed herbicidal potential against different weeds [17,18] and the target of these phytotoxins on plant cells are mitochondria, chloroplast, plasma membrane, Golgi complex and nucleus, etc. [3]. Tenuazonic acid (TeA) is one of the major toxic compounds produced by *Alternaria* species [19,20]. TeA shows also other biological properties including antitumor, antibacterial, antiviral, and phytotoxic activities [21,22]. It is also known for its broad-spectrum herbicidal activity to diverse speciessuch as *Digitariasanguinalis, Amaranthus retroflexus,* *Ageratina adenophora, Acalypha australis, Alopecurus japonicus, Ambrosia artemisiifolia, Commelina communis, Cyperus iria, C. difformis, Echinochloa crus-galli, Eclipta prostrata, Erigeron annuus, Geranium carolinianum, Leptochloa chinensis, Sonchus asper, Trifolium repens*, and *Vicia sativa* [23,24].

In our previous investigations, *A. alternata* (isolates X3, S1, C1) used in the present study were evaluated for their herbicidal potential towards their target weed hosts under controlled and field conditions [25,26]. Disease severity following application of the isolates on their targets reached up to 99% in the detached leaf assay, 80% in pot trial, and 43% in field experiments. The rapeseed-oil-based liquid formulation of isolate X3 reduced cocklebur (*Xanthium strumarium* L.) height, root length and dry weight by up to 50%, 27%, and 58%, respectively in pot trial and 40%, 51%, and 45%, respectively in the field experiment. Similarly, the liquid formulation of isolate S1 reduced sow thistle (*Sonchus oleraceus* L.) height, root length, and dry weight by up to 80%, 65%, and 80%, respectively in pot trial and 39%, 50%, and 44%, respectively in the field experiment [27] unpublished data. None of the crops tested for host specificity including tomato (*Solanum lycopersicum* cv. Saint Pierre, Solanaceae), wheat (*Triticum durum* cv. Vitron, Poaceae), faba bean (*Vicia faba* cv. Aquadulce, Fabaceae), and zucchini (*Cucurbita pepo* cv. Quarantaine, Cucurbitaceae) was susceptible to the three *A. alternata* isolates.

The aim of this study was to characterize, with a metabolomic approach, three different strains of *Alternaria alternata* (isolates X3, S1, C1) recovered from their target weeds—namely *Xanthium strumarium* L., *Sonchus oleraceus* L., and *Convolvulus arvensis* L.—and to evaluate their myco-herbicidal potential on detached leaves of their host weeds.

## 2. Results

### 2.1. Chemical Characterization of Alternaria alternata Isolates C1, S1, and X3

Organic crude extracts, obtained from *A. alternata* culture filtrates, showed differences in terms of biochemical profile. Hence, the number of compounds detected in the organic extracts was 27, 29, 20 compounds (identified and unidentified), respectively in *A. alternata* C1, S1, and X3. LC-MS qTOF analysis allowed the identification of 15 compounds differently occurring in the isolates. In Table 1 and (Supplementary Materials) Table S1 are reported the main compounds detected on the crude extract of C1, S1, and X3.

**Figure 1:**
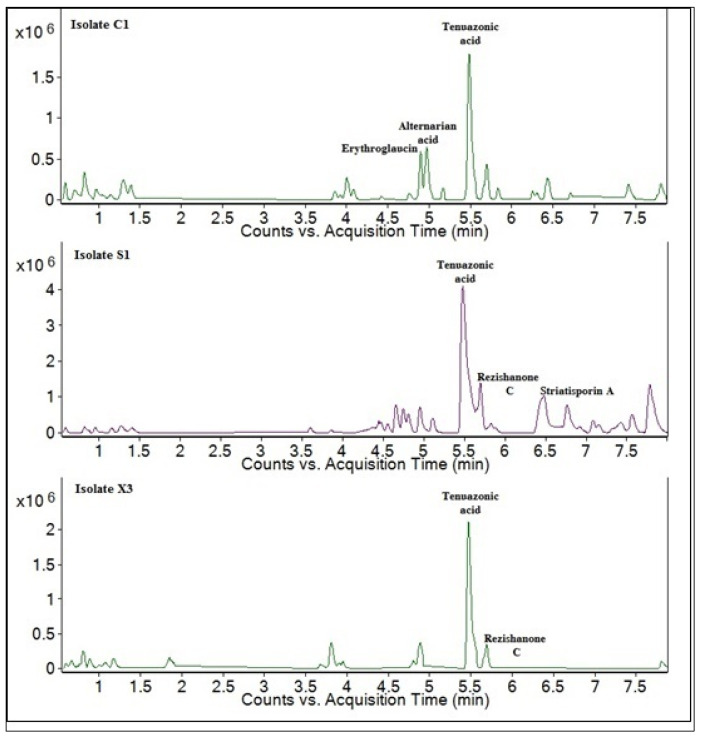
Total ion chromatograms (TIC) LC-MS qTOF chromatograms of *Alternaria alternata* organic extracts of isolates X3, S1, and C1.

Figure 1 shows total ion chromatograms (TIC) for the three isolates. It can be seen that tenuazonic acid (TeA) represents the major compound in organic extracts of all the three isolates in an isolate-dependent concentration (relative concentration).

Cyclo-L-prolylglycine, verrol, brassicene F, and rezishanone C were also detected in all samples at a lower concentration compared to TeA. Brassicicene D is another common compound, produced by *A. alternata* isolates C1 and S1. The compounds 12-methoxycitromycin, erythroglaucin, alternarian acid, TAN 913, chaetoquadrin E, and tanzawaic acid F are compounds only found in the culture filtrate of isolate C1, whereas cytosporin C and striatisporin A are only found in the culture filtrate of *A. alternata* isolate S1. Similarly, Cyclo-(Pro-Ala) is only found in the culture filtrate of *A. alternata* isolate X3 (Table 1).

The analysis of the samples obtained after fractionating the crude extract of C1 showed that all fractions contain the *Stachybotrys microspora* metabolite named triprenylphenol-7 (SMTP-7). The fractions from C1 contained also tenuazonic acid, decarestrictin N that was less abundant, and only one fraction contained tricycloalternarene (ACTG) toxin A.

Regarding *A. alternata* isolate S1, 2 out of 7 fractions—obtained from the crude extract—contain SMTP-7 and tenuazonic acid; while ACTG toxin A (low abundance), virescenoside M and phoenistatin (high abundance) were detected in one fraction.

As for the composition of *A. alternata* isolate X3, 6 out of 8 fractions contain decarestrictin N, while all the fractions contain SMTP-7. Five out of the 8 fractions contain tenuazonic acid and benzoic acid is detected in two fractions in small amounts. Cyclo-(L-Phe-L-Pro) is found exclusively in one fraction of isolate X3 (Supplementary Materials: Table S2).

### 2.2. Herbicidal Activity (Detached Leaf Assay)

#### 2.2.1. Effect of C1 Metabolites on Detached Leaves of *Convolvulus arvensis*

**Figure 2:**
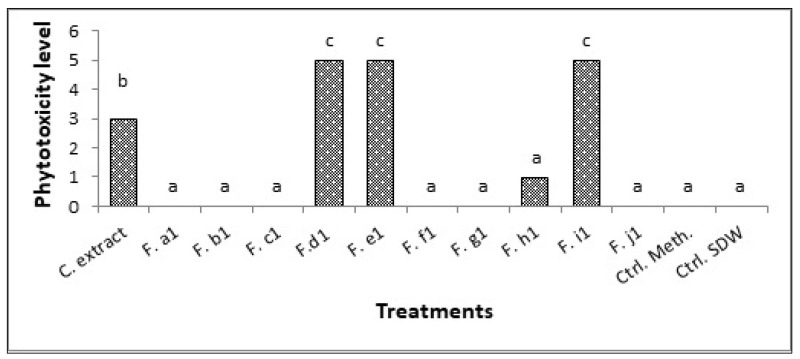
Effect of exometabolites of *Alternaria alternata* isolate C1 on injured detached leaves of *Convolvulus arvensis*. C. extract: crude extract; F.a1–j1: Fractions a1–j1; Ctrl. Meth.: methanol control; Ctrl. SDW: sterile distilled water control; a, b, c: represent the homogeneous groups.

Fractions d_1_, e_1_, h_1_, and i_1_ were able to cause symptoms on injured detached leaves. Analysis of variance showed a significant effect of the crude extract and the fractions d_1_, e_1_, and i_1_ on the severity of phytotoxicity on injured leaves (*p* ≤ 0.001, df = 12, MS = 5782.59, F = 346.95) (Figure 2).

**Figure 3:**
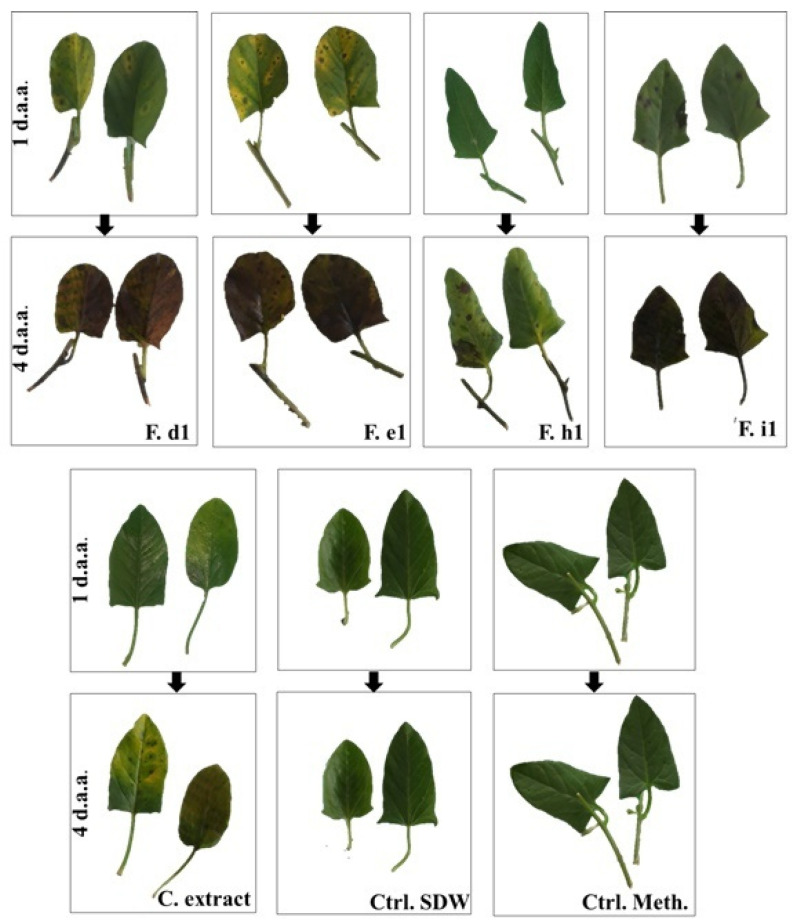
Appearance of the injured detached leaves of *Convolvulus arvensis* after treatment with the crude extract and fractions d_1_, e_1_, h_1_, and i_1_ of *Alternaria alternata* isolate C1. d.a.a.: days after application (1 or 4 days). C. extract: crude extract; F.a1–j1: Fractions a1–j1; Ctrl. Meth.: methanol control; Ctrl. SDW: sterile distilled water control.

C1 crude extract and d_1_–e_1_ fractions resulted in small necroses surrounded by chlorosis; both fractions caused total leaf destruction at the end of the experiment (4 days). The h_1_ fraction caused larger necrosis compared to d_1_ and e_1_ but did not result in total leaf destruction; the i_1_ fraction caused black spots that subsequently occupied the entire leaf surface. Both controls remained symptomless (Figure 3).

#### 2.2.2. Effect of S1 Metabolites on Detached Leaves of *Sonchus oleraceus*

**Figure 4:**
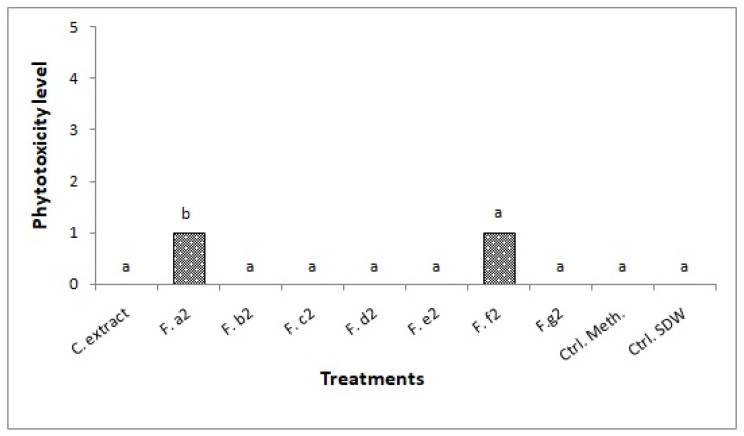
Effect of crude extract and fractions of *Alternaria alternata* isolate S1 on injured detached leaves of *Sonchus oleraceus*. C. extract: crude extract; F. a2–g2: Fractions a2–g2; Ctrl. Meth.: methanol control, Ctrl. SDW: sterile distilled water control; a, b: represent the homogeneous groups.

Fractions a_2_ and f_2_ caused symptoms on the injured detached leaves. The analysis of variance showed a significant phytotoxic effect of fraction a_2_ on the injured detached leaves (*p* = 0.001, df = 9, MS = 124.44, F = 14.93). However, severity level of both fractions was not high (Figure 4).

**Figure 5:**
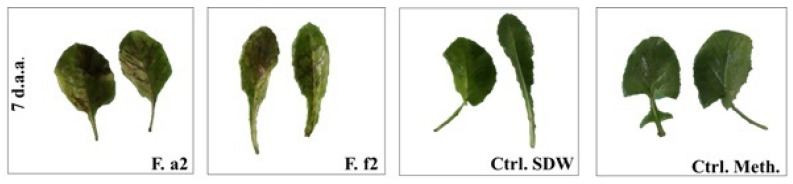
Appearance of the injured detached leaves of *Sonchus oleraceus* after treatment with the fractions a_2_ and f_2_ of *Alternaria alternata* isolate S1. d.a.a.: days after application. F.a2–f2: Fractions a2–f2; Ctrl. Meth.: methanol control, Ctrl. SDW: sterile distilled water control.

Symptoms caused by both fractions were in the form of brown areas (Figure 5). The crude extract did not cause any symptoms.

#### 2.2.3. Effect of X3 Metabolites on Detached Leaves of *Xanthium strumarium*

The crude extract and the eight fractions recovered from *A. alternata* isolate X3 did not show any effect on either injured or uninjured detached leaves of the target weed.

Table 2 shows the active fractions and their corresponding exometabolites identified by LC-MS analysis. The main compounds common in all the active fractions are SMTP-7 and tenuazonic acid.

## 3. Discussion

The biochemical profile of organic extracts of the three isolates of *A. alternata* showed different composition, but with some common compounds—i.e., tenuazonic acid and SMT7 that were found in almost all fractions from the three crude extracts. These results are in agreement with those reported by Sottoret al. [28]. Their work showed that *Streptomyces* strains, closely related through the 16S rRNA gene marker, produced a common set of natural compounds and another set that is unique for each strain (SN25_8.1 and DSM 40236T); they considered the common secondary metabolites as core molecules and the strain-specific compounds as accessory natural products. Ukaet al. [29] also found that *Aspergillus flavus* has a high intraspecies diversity of secondary metabolites. Llorenset al. [30] studied the influence of incubation temperature, water activity (aw), and type of isolate on the production of deoxynivalenol (DON), nivalenol (NIV), and 3-acetyldeoxynivalenol (3-AcDON) by three isolates of *Fusarium graminearum* and three isolates of *Fusarium culmorum* from different hosts (wheat, corn, banana, and leek) in different regions of Spain. They found that the production of these toxins differs (present, absent, and trace) between isolates of the same species grown in the same conditions.

Davis et al. [17], found that isolates 582 and 584 of *Alternaria alternata* produced tenuazonic acid but not isolate 938 of the same fungal species when grown on cottonseed and yeast-sucrose extract broth.

Schumacher et al. [31] also found a difference in biochemical composition of seven strains of *A. alternata* isolated from plant litter from different regions in Australia.

This difference among strains can be explained by morphological and cultural characteristics Schumacher et al. [31], the horizontal genes transfer (HGT) of entire biosynthetic pathways [32,33], the environmental competition, the survival needs of the strain, the need to adapt and specialize in their ecological niche, and host differences [28,29].

In our study, tenuazonic acid was found to be the major compound of the three culture filtrates and was detected in most of the fractions. This finding agrees with results of Meronucket al. and Davis et al. [17,34] that found this metabolite in their selected strains at different concentrations. Tenuazonic acid was first isolated from *Alternaria tenuis* by Rosettet al. [35]. It was detected in culture filtrates of several species of *Alternaria* genus: *A. longiceps*, *A. kikuchiana*, *A. mali*, *A. alternata*, and *A. tenuissima* [19,36]. Tenuazonic acid is known as a natural potential herbicide for which different bioassays (such as cut shoot, seedling, and detached leaf bioassays) have been used [16,18].

The second important exometabolite detected was SMTP-7 (*Stachybotrys microspora* triprenyl phenol-7) which is a low molecular weight compound that has a thrombolytic, anti-inflammatory, and antioxidant effects. This molecule is usually secreted by the fungal species *Stachybotrys microspora* and is known for its therapeutic activity against cerebral infarction in several rodent models [36,37]. To the best of our knowledge, neither SMTP-7 nor 12-methoxycitromycin detected in the culture filtrate of isolate C1 (Table 2) have been reported to be produced by *Alternaria* spp.

Some fractions from isolates C1 and S1 (d_1_, e_1_, h_1_, i_1_, a_2_, and f_2_) caused symptoms (necrosis and chlorosis) on injured detached leaves of *S. oleraceus* and *C. arvensis*. The four fractions (d_1_, e_1_, h_1_, and i_1_) that gave an effect on injured detached leaves of *C. arvensis* all contain SMTP-7 and tenuazonic acid. These compounds are also present in two other fractions (g_1_ and j_1_) that did not give an effect. Fractions a_2_ and f_2,_ that showed an effect on injured detached leaves of *S. oleraceus,* contain SMTP-7, whereas the fraction f_2_ contains tenuazonic acid and decarestrictin N. These identified compounds are also present in other fractions that did not give an effect. Therefore, phytotoxic activity might eventually be also explained by the synergistic effect of the different molecules identified in the fractions.

Our findings regarding the herbicidal effect agree with the results of several studies that showed the efficiency of secondary metabolites from *Alternaria* towards different weeds (*Xanthium occidentale*, *Parthenium hysterophorus*, and *Lantana camara*) [15,16,18]. Such effects may be due to the presence of phytotoxic substances with herbicidal potential [10,38,39].

The crude extract and its fractions from isolate X3 did not show any effect on the injured detached leaves of *X. strumarium*. This result could be explained if we consider that this weed is less sensitive to the tested compounds than the other two weeds. On the other hand, the toxicogenic potency of *Alternaria* species and the effect of each toxic component vary between different isolates [40,41]. Zhou et al. [24] found that the effect of secondary metabolites of *A. alternata* on plants increases with increasing concentration of secondary metabolites and that this effect differs between the plant species.

Secondary metabolites cannot penetrate the leaf, and for this reason several authors [15,38] recommended the combination of secondary metabolites with the pathogen itself (the pathogen could be assisted by the toxin during the penetration and colonization phase to increase the level of disease on the weed without any effect on the non-host plant), as well as with other control methods in integrated weed management.

## 4. Materials and Methods

### 4.1. Fungal Isolates

Three isolates of *Alternaria alternata* (Fries.) Kiessler (X3, S1, C1) were previously recovered from *Xanthium strumarium*, *Sonchus oleraceus* and *Convolvulus arvensis,* respectively and documented [25,42]. These isolates showed morphological and cultural differences. Sequences of internal transcribed spacer (ITS) of the isolates X3, S1, and C1 were deposited in the NCBI Genebank (accession numbers MH827056, MH879766, and MH828350) and showed 99% homology with the sequences of the holotype of *A. alternata* CBS 916.96 (accession no. KF465761).

The fungal isolates were cultured on Potato Dextrose Agar medium (PDA; 200 g potato, 20 g dextrose, and 20 g agar in 1000 mL distilled water) in Petri dishes (9 cm in diameter) and incubated at 25 °C in the dark for 7 days, then stored at 4 °C until use.

### 4.2. Metabolites Production and Extraction

Small plugs (3 mm in diameter) from 7-day-old cultures of each of the fungal isolates were used to inoculate individually 2000 mL of Potato Dextrose Broth (PDB) liquid medium in 5000 mL capacity Erlenmeyer flasks. The fungal cultures were incubated statically under laboratory conditions for 21 days. Afterwards, mycelium-free culture filtrates were recovered by filtration through two-layer muslin cloth. The culture filtrates were subsequently extracted three times with equal volumes of ethyl acetate using a separating funnel and the resulting organic extracts were dehydrated with anhydrous sodium sulfate (Na_2_SO_4_). The extracts were concentrated using a rotary evaporator (IKA^®^-Werke GmbH & CO. KG, Staufen, Germany) under vacuum and at 40 °C [43].

### 4.3. Metabolite Fractionation

The crude extracts of isolates X3, S1, and C1 of *A. alternata* were fractionated by direct phase column chromatography (CC) eluted with different solvents (Supplementary Materials: Table S3) yielding different groups of homogeneous fractions.

### 4.4. HPLC-MS Analysis

A high-performance liquid chromatography-mass spectrometry (HPLC-MS) method was developed using an HPLC coupled to a quadrupole time-of-flight (Q-TOF) mass spectrometer with a dual electrospray ionization (ESI) source and equipped with a diode array detector (DAD) system (Agilent Technologies, Santa Clara, CA). Flow rate was set at 0.40 mL min^−1^. Metabolites were eluted at constant temperature of 37 °C, using a linear gradient composed by A: 0.1% (v/v) formic acid (FA) in H_2_O and, B: 0.1% (v/v) FA in acetonitrile (ACN). The gradient was as follows: starting condition 5% B, 0 min, 100% B in 6 min and held for8 min, 5% B for 9 and 10 min. UV spectra were collected by DAD every 0.4 s from 190 to 750 nm with a resolution of 2 nm. MS parameters were set with Agilent Mass Hunter Data Acquisition Software, rev. B.05.01. The instrument operated in positive mode, as [M + H]^+^ions; MS spectra were recorded in centroid mode, with an *m/z* 100–1700 mass range. The injected sample volume was 7 μL. In order to perform real-time lock mass correction, an isocratic pump (1260 Infinity Series, Agilent Technologies) was used to infuse a standard solution consisting of two reference mass compounds: purine (C_5_H_4_N_4_, *m/z* 121.050873, 10 μmolL^−1^) and hexakis (1H,1H,3H-tetrafluoropentoxy)-phosphazene(C_18_H_18_O_6_N_3_P_3_F_24_, *m/z* 922.009798, 2 μmolL^−1^). Flow rate was set at 0.06 mL min^−1^ while the detection window and the minimum height were set at 1000 ppm and 10,000 counts, respectively, for reference mass correction.

### 4.5. Detached Leaf Assay

Healthy leaves detached from the three weed hosts were surface sterilized in 2% sodium hypochlorite for 5 min, rinsed three times with sterile distilled water (SDW) and blotted dry in sterile paper sheets. Half of the leaves were injured using fine sterile needles (10small wounds were practiced over the entire surface of the leaf blade). Injured and uninjured leaves were then placed adaxial side up on wet filter paper in glass sterile Petri dishes (9 cm diameter, 2 leaves per dish).

Leaves were then treated each with 50 µL of the crude extract solutions and their fractions applied separately all over the limb. To distinguish between fractions, the numbers 1, 2, and 3 were assigned, respectively to fractions from isolates C1, S1, and X3. The fractions; c_1_, e_2_, e_3_, and d_3_ were not tested because of insufficient amounts. In the injured leaves, 5 µL of each diluted fraction were deposited on the wounds. Control leaves were treated with the same volume of SDW or with methanol at 2% [15,18]. Crude extracts and fractions were used at a concentration of 0.1 mg mL^−1^. All leaves were incubated at 25 °C and 60% relative humidity (RH) under laboratory conditions. Phytotoxic effect was monitored daily starting from the first day after treatment. At the end of the experiment which lasted 4, 7, and 9 days for the detached leaves of *C. arvensis*, *S. oleraceus*, and *X. strumarium* respectively, the severity of phytotoxicity of crude extracts and fractions tested was recorded for each treatment using a 0–5 rating scale of [16] as follows:0: 0–4%: no effect, 1: 2–19%: slight chlorosis; 2: 20–49%: marked chlorosis and slight necrosis; 3: 50–79%: acute chlorosis and marked necrosis; 4: 80–94%: high chlorosis and high necrosis; 5: 95–100%: acute chlorosis and necrosis.

### 4.6. Data Analysis

Detached leaf experiment was conducted in a randomized complete block design and was repeated twice with 2 replicates per treatment in the first experiment and 3 replicates in the second one. Data were subjected to analysis of variance (One-way ANOVA). Means were compared using HSD of Tukey at *p* ≤ 0.05 using the STATISTICA software (Statistica version 8.5, year 2014).

## 5. Conclusions

It can be concluded from our findings that the biochemical composition of the organic extracts showed great biochemical heterogeneity within the three isolates (C1, S1, and X3) of *A. alternata* with the remarkable presence of tenuazonic acid and SMTP-7. Only the crude extract of isolate C1 and some fractions (d_1_, e_1_, h_1_, i_1_, a_2_, and f_2_) of the two isolates (C1 and S1) of *A. alternata* showed symptoms (necrosis and chlorosis) on the injured detached leaves of *C. arvensis* and *S. oleraceus*, respectively. Therefore, it would be interesting to broaden the spectrum of research to highlight the whole biochemical active composition of these fungi and their effects on other weeds.

## Figures and Tables

**Table 1:** Main secondary metabolites identified in organic extracts of *Alternaria alternata* C1, S1, and X3 by LC-MSqTOF analysis.

Compounds	*Alternaria alternata* Isolates		
	C1	S1	X3
Cyclo-l-prolylglycine	+	+	+
12-Methoxycitromycin	+	−	−
Erythroglaucin	+	−	−
Alternarian acid	+	−	−
TAN 913	+	−	−
Verrol	+	+	+
Tenuazonicacid	+	+	+
Brassicicene F	+	+	+
Rezishanone C	+	+	+
Brassicicene D	+	+	−
Chaetoquadrin E	+	−	−
Tanzawaicacid F	+	−	−
Cytosporin C	−	+	−
Striatisporin	−	+	−
Cyclo-(Pro-Ala)	−	−	+
Triprenyl phenol-7 (SMTP-7)	+	+	+

+ = presence of the specific compound;− = absence of the specific compound.

**Table 2:** Active fractions and corresponding exometabolites identified.

Alternaria alternataIsolates	Active Fractions	Compounds
*A. alternata* C1	d_1_	SMTP-7 (+)-Phomopsidin Tenuazonic acid
	e_1_	Decarestrictin N SMTP-7 Tenuazonic acid
	h_1_	SMTP-7 Tenuazonic acid
	i_1_	Decarestrictin N SMTP-7 Tenuazonic acid
*A. alternata* S1	a_2_	SMTP-7
	f_2_	Decarestrictin N SMTP-7 Tenuazonic acid

## Data Availability

The datasets generated during and/or analysed during the current study can be find in the main text and the supplementary materials.

## Supplementary Materials

The following are available online at https://www.mdpi.com/article/10.3390/pathogens10111448/s1, Table S1: Putatively identified metabolites in cultural filtrates of *A. Alternata* isolates C1. S1 and X3 obtained by LC-MS analysis. Data include retention time, experimental and theoretical mono-isotopic mass and molecular formula, Table S2: Chemical characterization of *Alternaria alternata* fractions (isolates C1. S1 and X3).(+ indicate the presence of a single compound; − indicate the absence of the single compound), Table S3: Solvent proportions used as eluents in the fractionation of crude extracts of *Alternaria alternata* isolates X3 S1 and C1 by column chromatography.
