# Supplementary material for: Metabolic Profile and Mycoherbicidal Activity of Three Alternaria alternata Isolates for the Control of Convolvulus arvensis, Sonchus oleraceus, and Xanthium strumarium
Source: Pathogens. 2021 Nov 7;10(11):1448. doi: 10.3390/pathogens10111448 (PMC8620798; doi:10.3390/pathogens10111448)
Supplement: Supplementary file 1 [file pathogens-10-01448-s001.zip › pathogens-1429271-supplementary.pdf]

Article

Supplementary Materials：Phytotoxic Secondary Metabolites from Three *Alternaria alternata* Isolates

NesmaAbdessemed [1]. Alessia Staropoli [2,3]. NadjiaZermane [4] and Francesco Vinale [3,5,*]

TableS1. Putatively identified metabolites in cultural filtrates of *A. Alternata* isolates C1. S1 and X3. obtained by LC-MS analysis. Data include retention time. experimental and theoretical mono-isotopic mass and molecular formula.

Compound	Retention time (min)	Experimental monoisotopic mass (Da)	Theoretical monoisotopic mass (Da)	Molecular formula
Cyclo-l-Prolylglycine	0.956	154.0746	154.0742	$C_7H_{10}N_2O_2$
12-Methoxycitromycin	4.895	260.0697	260.0684	$C_{14}H_{12}O_5$
Erythroglaucin	4.900	300.062	260.0684	$C_{16}H_{12}O_6$
Alternarian acid	4.970	320.0548	320.0532	$C_{15}H_{12}O_8$
TAN 913	4.971	302.0443	302.0426	$C_{15}H_{10}O_7$
Verrol	5.456	378.2051	378.2042	$C_{21}H_{30}O_6$
Tenuazonic acid	5.490	197.106	197.1051	$C_{10}H_{15}NO_3$
Brassicicene F	5.537	364.2261	364.2249	$C_{21}H_{32}O_5$
Rezishanone C	5.698	320.1652	320.1623	$C_{18}H_{24}O_5$
Brassicicene D	5.833	362.2102	362.2093	$C_{21}H_{30}O_5$
Chaetoquadrin E	6.710	322.1404	322.1416	$C_{17}H_{22}O_6$
Tanzawaic acid F	7.412	304.169	304.1674	$C_{18}H_{24}O_4$
Cytosporin C	6.460	310.1797	310.1780	$C_{17}H_{26}O_5$
Striatisporin	6.461	254.1165	254.1154	$C_{13}H_{18}O_5$
Cyclo-(Pro-Ala)	3.821	226.1321	226.1317	$C_{11}H_{18}N_2O_3$
SMTP-7	4,786	868,489	868.4873	$C_{51}H_{68}N_2O_{10}$

Table S2. Chemical characterization of *Alternaria alternata* fractions (isolates C1. S1 and X3). (+ indicate the presence of a single compound; - indicate the absence of the single compound).

Compounds	Fraction a			Fraction b			Fraction c			Fraction d			Fraction e			Fraction f			Fraction g			Fraction h			Fraction i			Fraction j		
	C1	S1	X3	C1	S1	X3	C1	S1	X3	C1	S1	X3	C1	S1	X3	C1	S1	X3	C1	S1	X3	C1	S1	X3	C1	S1	X3	C1	S1	X3
Decarestrictin N	−	−	+	+	−	−	+	−	+	−	−	−	+	+	+	+	+	+	+	+	+	−	−	+	+	−	−	−	−	−
Cyclo-(L-Phe-L-Pro)	−	−	+	−	−	−	−	−	−	−	−	−	−	−	−	−	−	−	−	−	−	−	−	−	−	−	−	−	−	−
SMTP-7	−	+	+	+	−	+	+	+	+	+	+	+	+	+	+	+	+	+	+	+	−	+	−	+	+	−	−	+	−	−
Tenuazonic acid	−	−	+	+	+	+	−	+	−	−	+	−	−	+	−	+	+	+	+	+	+	+	−	+	+	−	−	+	−	−
2-(4-Hydro+yazobenzene)	−	−	+	+	−	+	−	−	−	−	−	−	−	−	−	−	−	−	−	−	−	−	−	−	−	−	−	−	−	−
Benzoic acid	−	−	+	+	−	+	−	−	−	−	−	−	−	−	−	−	−	−	−	−	−	−	−	−	−	−	−	−	−	−
(+)-Phomopsidin	−	−	−	+	−	−	−	−	−	+	−	−	+	−	−	−	−	−	+	−	−	−	−	−	−	−	−	−	−	−
Virescenoside M	−	−	−	−	+	−	−	−	−	−	−	−	−	−	−	−	−	−	−	−	−	−	−	−	−	−	−	−	−	−
Phoenistatin	−	−	−	−	+	−	−	−	−	−	−	−	−	−	−	−	−	−	−	−	−	−	−	−	−	−	−	−	−	−
ACTG to+in A	−	−	−	−	+	−	−	−	−	−	−	−	−	−	−	−	−	−	+	−	−	−	−	−	−	−	−	−	−	−
Wortmannilactones (F-H)	−	−	−	−	−	−	−	−	+	−	−	−	−	−	−	−	−	−	−	−	−	−	−	−	−	−	−	−	−	−
Fumagillin methyl ester	−	−	−	−	−	−	−	−	+	−	−	−	−	−	−	−	−	−	−	−	−	−	−	−	−	−	−	−	−	−
Viridominic acid C	−	−	−	−	−	−	−	−	+	−	−	−	−	−	−	−	−	−	−	−	−	−	−	−	−	−	−	−	−	−
Botrydial	−	−	−	−	−	−	−	−	−	−	−	+	−	−	−	−	−	−	−	−	−	−	−	−	−	−	−	−	−	−
Ganoderic acid B	−	−	−	−	−	−	−	−	−	−	−	−	−	−	+	−	−	−	−	−	−	−	−	−	−	−	−	−	−	−
Viridominic acid B	−	−	−	−	−	−	−	−	−	−	−	−	−	−	+	−	−	−	−	−	−	−	−	−	−	−	−	−	−	−
Picolinic acid	−	−	−	−	−	−	−	−	−	−	−	−	−	−	−	−	−	−	−	−	−	−	−	+	−	−	−	−	−	−

Table S3. Solvent proportions used as eluents in the fractionation of crude extracts of *Alternaria alternata* isolates X3. S1 and C1 by column chromatography.

**Eluents used for fractionation**	**Volume (ml)**	***Alternariaalternata* isolates**		
		**X3**	**S1**	**C1**
Chloroform/methanol (95:5)	100	x	x	
Chloroform/methanol (90:10)	100	x	x	
Chloroform/methanol (80:20)	100	x	x	
Methanol	50	x	x	
Ethyl acetate/petroleum ether (1:9)	50			x
Ethyl acetate/petroleum ether (2:8)	50			x
Ethyl acetate/petroleum ether (1:1)	100			x
Ethyl acetate/petroleum ether (8:2)	100			x
Ethyl acetate/petroleum ether (9:1)	100			x
Ethyl acetate	100			x
Methanol	100			x
